# Socio-emotional and personal development competencies as assets facilitating psychosocial adaptation in socially vulnerable secondary school students

**DOI:** 10.3389/fpsyg.2025.1462605

**Published:** 2025-04-30

**Authors:** Victor Manuel Pardo, Susana De La Ossa, Manuel Noreña

**Affiliations:** ^1^Psychology Program, University of San Buenaventura, Cartagena, Colombia; ^2^Department of Educational Studies, School of Arts and Humanities, University of Caldas, Manizales, Colombia

**Keywords:** developmental assets, psychosocial adjustment, adolescence, social vulnerability, wellbeing

## Abstract

Fostering positive developmental assets in the school student population could improve individuals' psychological adjustment processes and personal wellbeing. In view of the above, employing a non-probability selective, cross-sectional design, this study evaluates the association between socio-emotional and personal development competencies and psychosocial adaptation in a sample of 169 socially vulnerable secondary school students in the city of Cartagena de Indias (Colombia). Of these participants, 53.3% were female, and ages ranged from 11 to 19 years (*M* = 14.2; *SD* = 1.9). Participants completed scales that assessed their personal development (self-esteem, optimism, and satisfaction with life) and their socio-emotional development (empathy, attention, emotional clarity, and repair), as well as a psychosocial adaptation scale. Once data were processed through one-factor analysis of variance and multiple correspondence analysis (MCA), it was found that socio-emotional development assets associated with emotional intelligence had a significant relationship with the psychosocial adaptation processes of adolescents, despite the disadvantages, and inequalities of the social environment in which they grew. Further studies with adolescent populations in this social context are required to confirm these findings and, eventually, create psychoeducational programs that promote these positive developmental assets.

## 1 Introduction

Research has claimed that fostering youth development represents an important paradigm shift (Benson et al., 2013), despite the predominance of deficit-oriented models of adolescent development. On this basis, a new model emerged, focused on positive development incorporating notions such as psychological wellbeing, personal initiative, and social competence as indicators of good development at particular stages of the life cycle (Barrios and Frías, 2016).

This new model considers that although adolescence is a part of evolutionary development, it cannot be defined solely based on the problems arising from the interaction between individuals and their environment. Conversely, it is necessary to understand the growth of adolescents within the framework of health to highlight positive resources during individuals' development. Therefore, it is crucial to specify indicators concerning mental health, behavioral adjustment, and relational and social efficiency that allow us to consider young people as individuals with potential to develop rather than problems to be solved (Oliva, 2004).

In recent decades, socio-emotional and personal development competencies have been consolidated as an essential pillar for academic, personal, and professional success (Lin et al., 2024). These competencies have received various labels, such as non-cognitive factors (Heckman et al., 2006) and are often associated with related constructs such as emotional intelligence (Salovey and Mayer, 1990), social and behavioral skills (DiPrete and Jennings, 2012), and interpersonal sensitivity (Murphy and Hall, 2011). Although definitions may vary, there is consensus that these competencies encompass a set of basic skills that are essential assets within the framework of positive youth development (Oliva, 2004; Benson et al., 2013). Based on this definition, adolescent positive development requires programs, organizations, and communities to build on young people's strengths to promote their health and wellbeing; further, favorable conditions should be created to nurture the social competencies, behaviors, and skills essential for success in adolescents' social, academic, and professional lives while promoting the prevention of risky behaviors (Aránzazu et al., 2021). Two key elements of this approach are (1) the idea of “plasticity” as every young person's ability to transform their relationship with their environment, prevent problems, and maximize their possibilities despite their individual or contextual characteristics (Lerner et al., 2021) and (2) the concept of assets for development (developmental assets), which include the resources of an individual, their family, school, or community that are essential for promoting positive adolescent development (Benson et al., 2013). These assets can also be understood to constitute adolescents' ability to respond in a flexible and adaptive manner to the evolutionary demands they must face throughout their development.

Another point of discussion regarding positive adolescent development relates to the process of adaptation and psychosocial adjustment. These factors determine how successfully young people can face the normative changes or crises that they experience during their transition to adulthood. Adaptation is a term used by Roy (2009) to define the process through which people become aware of their thoughts and emotions during their interactions with others and the environment and the result of this awareness. This term is also a key element in Levine's conservation model, where it is assumed to be a product of the interactions between individuals and the environment (Londono and McMillan, 2015).

Later, Gómez-Ramírez and De la Iglesia (2017) suggested that adaptation is an individual's response to changes or conflicts caused by internal (such as food, rest, or medical condition) or external factors as they learn about their needs. Therefore, a proper adaptation process requires acknowledging one's emotions and developing individuals' skills in building appropriate personal, family, and non-family relationships. In this connection, psychosocial adjustment in adolescents requires emotional intelligence (EI) and basic skills for appropriate emotional processing (Palomera et al., 2012). Recent studies have found that EI can directly influence adolescent development both within and outside the school context; thus, a lack of EI among adolescents may create problems related to psychological adjustment, quality of interpersonal relationships, wellbeing, and academic performance, among others (Fernández-Berrocal et al., 2022). Intelligent behavior is essentially adaptive, in that it represents effective ways of responding to contextual demands (Barraza-López et al., 2017).

Psychosocial adaptation in the positive development approach also implies acknowledging environments, or, in other words, the conditions, circumstances, and influences affecting adolescents and their behavior (Alvarado-García et al., 2023). In this regard, it has been shown that socially disadvantaged environments may lead to behavioral problems in children and adolescents for various reasons, ranging from socioeconomic to psychological factors. Factors such as economic insecurity, a lack or poor quality of basic resources, and violence in the community, in addition to other social problems, increase the risk of behavioral, mental, and psychosocial adjustment disorders. Although the literature has not fully identified the variables that determine the adaptation of young people in vulnerable social contexts—given that these variables may vary significantly (Neely-Prado et al., 2021)—it has demonstrated that growing up in poverty, as indicated by low socioeconomic status, can serve as a predictor of emotional difficulties, behavioral problems, academic performance challenges, and overall mental health issues in young people (Yoshikawa et al., 2012; Kim et al., 2013; Neely-Prado et al., 2019).

In this regard, Heckman et al. (2006) argues that the importance of socio-emotional and personal development skills is accentuated in contexts of social vulnerability, where the lack or poor development of non-cognitive skills is associated with the emergence of risk behaviors, such as antisocial behavior, substance use, and involvement in illegal activities. In this sense, quality education in the early years can play a key role in fostering these skills, even among the most disadvantaged youth. Heckman (2008) perspective reinforces this idea, emphasizing that the formation of skills throughout the life cycle is a dynamic process in which early investments in vulnerable children and youth increase the productivity of later investments. This approach promotes not only educational success and psychosocial adjustment, but also long-term social and economic integration. Similarly, Heckman et al. (2006) underscore the critical role of the family in shaping adult outcomes. Investing in the early development of disadvantaged children can reduce the achievement gap and improve social and economic wellbeing throughout life, demonstrating not only the predictive power of non-cognitive skills such as perseverance and self-confidence, but also their relevance at the level of cognitive skills for labor market success and social adjustment.

Some studies have examined the relationship between psychosocial adjustment in vulnerable populations and socio-affective, cognitive, and behavioral variables, while others have emphasized the influence of clinical and pathological conditions on psychosocial adjustment (Neely-Prado et al., 2021; Orth and Robins, 2014; Prati and Pietrantoni, 2009; Bartels and Pratt, 2009). In terms of developmental assets, correspondences have been found between the characteristics of families (Parra and Oliva, 2015), school (Pertegal and Hernando, 2015; Oliva et al., 2008), community and neighborhood with the positive development of adolescents (Oliva and Antolín-Suárez, 2015). In this sense, it has been concluded that a warm and safe climate, connection with the school, clear rules and the possibility to participate in school activities can promote students' attachment to school, wellbeing and psychological adjustment. Similarly, parental affection, communication, control, and supervision, mediated by positive parenting practices, can promote adolescents' autonomy, adjustment, and social competence. Community and broader community assets, such as safety, availability of extracurricular activities, empowerment, and community support for young people, are essential in promoting positive youth development (Leventhal et al., 2009). Studies also link community assets to the concept of social capital, whereby young people's desire to participate in the community is determined by their sense of belonging to the place where they live (Cancino, 2005; Oliva et al., 2012).

In these contexts, researchers have also been interested in understanding the influence of peers, friendships and social networks as assets that can support adolescents' psychological, social, and emotional wellbeing and self-esteem (Shapiro and Margolin, 2014). Others have analyzed the relationship between adolescents' developmental assets, school adjustment (satisfaction with school), and life satisfaction (Gutiérrez and Gonçalves, 2013). The literature on developmental assets, psychosocial adjustment, and adaptation may be consistent with the concept of positive development. However, there is limited research on how managing developmental assets can foster adaptive responses and psychosocial adjustment in adolescents from vulnerable social contexts. Therefore, the aim of the present study was to empirically investigate the relationship between developmental assets and psychosocial adjustment in socially vulnerable youth. It was hypothesized that personal and socio-emotional developmental assets would facilitate adaptive responses and enhance psychosocial adjustment in adolescents from these contexts.

Within the framework of positive development, schools are presented as key spaces to promote and strengthen the wellbeing and mental health of young people. For this reason, the school must offer programs that foster the competencies and skills necessary to establish positive links between adolescents, their families and the community (Martín-Quintana et al., 2023). Therefore, it is essential to learn about the characteristics of the context that significantly contributes to the healthy development of its members from a health promotion perspective as adolescents who have properly developed can positively identify their identity and have adequate self-concept, healthy coping styles, appropriate norm introspection, and positive perceptions of family and social support (Oliva et al., 2010).

## 2 Materials and methods

### 2.1 Participants

This study was conducted in a public education institution (IEP for its Spanish acronym) in Ciudad Bicentenario, a neighborhood located in the southeastern area of the city of Cartagena, Colombia. It is regarded as a neighborhood of social interest that emerged as a macro urban project of the national government (Fox Pardo, 2014). It was developed as a dwelling solution for locating and relocating families from lower socioeconomic strata living in regions prone to natural hazards or belonging to high social-risk areas; families whose members reintegrated into civilian life (former combatants of illegal armed groups); or families displaced owing to the Colombian armed conflict (Carmona and Rodríguez, 2011).

Of the 315 students enrolled in secondary school at the Ciudad Bicentenario IEP, a non-probability convenience sample of 169 students (54% of the student population) was selected. Inclusion in the study required participants to meet the following criteria: provide written consent from their parents or guardians (for underage participants), complete an informed consent form, be currently enrolled in the school, and explicitly agree to participate in the study (both for underage and adult participants). The final sample, which included only students who met the requirements for informed consent and assent and completed all administered instruments, consisted of students in grades 7–11, with 90 females (53.3%) and 79 males (46.7%), ranging in age from 11 to 19 years (*M* = 14.2, *SD* = 1.9). No student from the final sample dropped out of the testing process.

### 2.2 Instruments

#### 2.2.1 Psycosocial adaptation

The Social Adaptation Self-Evaluation Scale (SASS) (Bosch et al., 1997) was used to measure psychosocial adaptation. This scale consists of 21 items that globally assess the subject's perception of their social functioning in areas such as behavioral strategies (“*Do you observe the social rules, good manner, politeness, etc.?*”), socio-family relationships (“*How frequently do you seek contacts with your Family*?”), socio-intellectual interests (“*Are you interested in scientific, technical or cultural information?*”), work, and leisure (“*Are you interested in hobbies/leisure*?”). The SASS is a Likert-type scale with four response levels ranging from 0 to 3, yielding a total score between 0 and 60 points. The first two items are mutually exclusive and are answered based on whether the respondent has a paid or unpaid occupation. The following ranges have been proposed as scoring criteria: 0–24 indicates social maladjustment, 25–52 falls within the normal range, and scores above 55 suggest “overadaptation,” considered pathological. The scale has demonstrated satisfactory internal consistency (α = 0.74) (Bosch et al., 1997). In the present study the reliability coefficient obtained (McDonald's Omega) was 0.86.

#### 2.2.2 Developmental assets

The parameters evaluated were as follows (Oliva et al., 2008, 2011): personal development (self-esteem, optimism, and satisfaction with life) and socio-emotional development (empathy, attention to emotions, and emotional clarity and repair).

##### 2.2.2.1 Self-esteem

This asset was evaluated using the Rosenberg Self-Esteem Scale (Rosenberg, 1965), a unidimensional, self-administered scale consisting of 10 items designed to assess the global self-esteem of adolescents (e.g., “*In general, I am satisfied with myself*,” “*I believe I have several good qualities*.”) Participants respond using a Likert scale ranging from 1 (Strongly disagree) to 4 (Strongly agree). While no official cut-off points are established for scoring, scores close to 40 suggest a positive perception of self-esteem, whereas scores near 10 indicate a negative self-perception. However, for the Colombian population, specific cut-off points have been proposed: for women, a score of 37 corresponds to the 75th percentile, and 30 to the 25th percentile; for men, the 75th percentile is represented by a score of 37 and the 25th percentile by a score of 31 (Gómez-Lugo et al., 2016). The reliability coefficient for this scale, McDonald's Omega, obtained in our study was 0.78

##### 2.2.2.2 Optimism

Optimism was assessed using the Spanish version of the General Mood subscale, part of the Emotional Quotient Inventory (EQ-i, YV) developed by Bar-On and Parker (2000). This subscale consists of eight items rated on a Likert scale ranging from 1 (Never) to 5 (Always), yielding a total score range of 8–40 points. Higher scores indicate greater optimism and improved functioning at both personal (better mood) and social levels. Some of its items explore statements such as: “*I am happy*” and “*In general, I expect the best*.” As cut-off points, scores corresponding to the 75th and 25th percentiles have been established for different age groups. For adolescents aged 14 to 15 years, the 75th percentile corresponds to a score of 37 for females and 39 for males, while the 25th percentile corresponds to scores of 28 for females and 30 for males. For individuals aged 16 years or older, the 75th percentile is represented by scores of 35 for females and 38 for males, and the 25th percentile by scores of 27 for females and 29 for males (Oliva et al., 2011). The reliability coefficient obtained in this study, McDonald's Omega, was 0.88.

##### 2.2.2.3 Satisfaction with life

The Student's Life Satisfaction Scale (Huebner, 1991) was used to evaluate this personal development asset. This scale provides a subjective assessment of adolescents' perception of their own life satisfaction, which is associated with their perceived psychological wellbeing (e.g., “My life is going well.”) It is a unidimensional Likert-type scale comprising seven items, with response options ranging from 1 (Completely disagree) to 7 (Completely agree). Of the seven items, items 2, 3, and 4 are reverse-scored. Total scores range from 7 to 49, with higher scores indicating greater psychological wellbeing and life satisfactio-n, whereas lower scores may suggest anxiety, depression, and reduced life satisfaction. As cut-off points, a score of 42 (for both males and females) corresponding to the 75th percentile is established for adolescents aged 14 to 16 years, while a score of 32 (for both males and females) represents the 25th percentile. For individuals aged 16 years and older, the 75th percentile corresponds to a score of 41 for females and 42 for males, while the 25th percentile corresponds to scores of 29 for females and 32 for males (Oliva et al., 2011). The obtained reliability coefficient was deemed satisfactory (ω = 0.73).

##### 2.2.2.4 Empathy

The Basic Empathy Scale (Jolliffe and Farrington, 2006) was used to measure empathy. This instrument consists of nine items divided into two dimensions: affective empathy (“*After spending time with a friend who is feeling sad for any reason, I usually end up feeling sad as well*”) and cognitive empathy (“*When someone is feeling depressed, I usually understand how they feel*.”) Scoring these dimensions allows for obtaining separate scores for each, as well as a total empathy score, the latter being used for the purposes of this research. The total empathy score ranges from 9 to 45 points, with participants responding to each item on a Likert scale from 1 (Strongly disagree) to 5 (Strongly agree). Higher scores are associated with greater psychological and behavioral adjustment in adolescents.

As cut-off points for the total score, a score of 39 for females and 35 for males, corresponding to the 75th percentile, has been established for adolescents aged 14 to 15 years, while scores of 33 for females and 28 for males correspond to the 25th percentile. For adolescents aged 16 years and older, the 75th percentile is represented by scores of 38 for females and 35 for males, while the 25th percentile corresponds to scores of 33 for females and 29 for males (Oliva et al., 2011). In the present study, the scale demonstrated a reliability coefficient of ω = 0.74.

##### 2.2.2.5 Expressing, managing, and acknowledging emotions

The TMMS-24 scale (Fernández-Berrocal et al., 2004), a brief Spanish version of the TMMS-48 (Trait Meta-Mood Scale−48, Salovey et al., 1995), was used to evaluate attention to emotions (“*I pay close attention to my feelings*”–perception–), emotional clarity (“*I can come to understand my feelings*”–understanding–), and emotional repair (“*When I feel sad, I reflect on the many pleasures life has to offer*”–regulation–). These three dimensions are assessed through 24 items, rated on a 5-point Likert-type scale ranging from 1 (Completely disagree) to 5 (Completely agree). Each dimension yields scores ranging from 8 to 40, serving as indicators of participants' subjective perception regarding attention to emotions, emotional clarity, and emotional repair, which collectively constitute intrapersonal emotional intelligence (EI).

Very high or low scores in the attention to emotions dimension require special consideration, as high scores may indicate problems associated with stress, depression, or poor social functioning. Conversely, low scores may reflect an extreme neglect of emotions, potentially impairing adequate social functioning. Similarly, the emotional clarity and emotional repair dimensions follow a parallel interpretation criterion: higher scores suggest proper emotional functioning and a tendency toward good mental health. The scores for each dimension of the TMMS-24 are classified and interpreted based on cut-off points derived from the scale's percentiles (Oliva et al., 2011; see Supplementary Table 1). The reliability coefficients obtained for each scale were as follows: Attention to emotions (ω = 0.86), Emotional clarity (ω = 0.88), and Emotional repair (ω = 0.81).

### 2.3 Procedures

Data collection was carried out following the approval of the research protocol by both the Bioethics Committee of Universidad de San Buenaventura Cartagena and the governing bodies (the rector and academic coordinators) of the Ciudad Bicentenario IEP. Parents and guardians were informed about the project through two methods: in person during special information meeting led by the rector and the school's social welfare team, and via written communication (informed consent) sent home with their children.

Participating students were informed of the objectives and scope of the research during a classroom visit conducted by psychology program students who were part of the formative research teams. These students facilitated the process of completing informed consent forms and collected the signed forms from parents or guardians.

The tests described above were administered in the classroom using a self-administered, paper-and-pencil format over two 45-min sessions. The students were grouped by academic level, resulting in a total of nine groups spanning grades 7 to 11. Each group was supervised during the data collection process by two pre-service research assistants.

The data collection process was carried out over a 3-month period, from August to October 2023, through 2 weekly sessions involving ~30 research assistants. The process was supervised by the authors of the study to ensure proper oversight and adherence to established research protocols.

### 2.4 Data analysis

The information was initially compiled in an Excel database and was later statistically processed through open-access data analysis programs such as R and JASP. One-factor analysis of variance (ANOVA) was used alongside JASP version 0.19.2 to independently evaluate the effect of each adolescent's positive developmental assets on their level of psychosocial adjustment. In addition, a multiple correspondence analysis (MCA) was conducted with the help of R version 4.2.3 and its libraries FactoMineR (Lê et al., 2008) and Factoextra (Kassambara and Mundt, 2020) to determine the patterns or profiles of association between the variables of interest in this study. Since all cases included in the final sample had complete responses, no data imputation procedures were required.

## 3 Results

A one-factor ANOVA was performed to examine the independent effects of each level of positive developmental resources on psychosocial adaptation within the study population. The analysis included the following developmental resources as independent variables: emotional repair, emotional clarity, emotional attention, optimism, self-esteem, satisfaction with life, and empathy. Each resource was treated as a categorical variable, classified into three levels (low, medium, and high) based on the 25th and 75th percentiles of their respective scores (see Supplementary material). The dependent variable was psychosocial adaptation, measured as a continuous score.

Table 1 summarizes the results of the ANOVA analyses, including *F-values*, degrees of freedom, *p-values*, and effect sizes (ω^2^). Significant effects were observed for emotional repair [F_(2, 166)_ = 27.3, *p* < 0.001, ω^2^ = 0.24], emotional clarity [*F*_(2, 84.2)_ = 25.6, *p* < 0.001, ω^2^ = 0.23], attention to emotions [*F*_(2, 78.1)_ = 27.9, *p* < 0.001, ω^2^ = 0.24], optimism [*F*_(2, 101)_ =10.4, *p* < 0.001, ω^2^ = 0.10], and self-esteem [*F*_(2, 58.8)_ = 3.99, *p* = 0.020, ω^2^ = 0.034]. In contrast, empathy [*F*_(2, 103)_ = 1.12, *p* = 0.314, ω^2^ = 0.002] and satisfaction with life [*F*_(2, 88.5)_ =1.27, *p* = 0.285, ω^2^ = 0.002] did not show significant effects.

**Table 1:** Analysis of variance (ANOVA), effects sizes and Levene's test of developmental assets.

**Variable**	***F***	***df1***	***df2***	***p***	***ω^2^***	**Levene *f***	***df1***	***df2***	***p***
Emotional repair	27.3	2	166	<0.001	0.24	1.13	2	166	0.325
Emotional clarity	25.6	2	84.2	<0.001	0.23	0.853	2	166	0.428
Attention to emotions	27.9	2	78.1	<0.001	0.24	2.20	2	166	0.115
Empathy	1.12	2	103	0.314	0.002	0.849	2	166	0.430
Self-esteem	3.99	2	58.8	0.020	0.034	1.10	2	166	0.335
Optimism	10.4	2	101	<0.001	0.10	0.513	2	166	0.600
Satisfaction with life	1.27	2	88.5	0.285	0.002	0.466	2	166	0.628

Effect size analysis indicates that emotional repair, emotional clarity, and attention to emotions had the strongest associations with psychosocial adaptation, each explaining 23%−24% of the variance in the dependent variable. Optimism exhibited a moderate effect (ω^2^ = 0.10), suggesting that it explains 10% of the variance. In contrast, self-esteem had a small but significant effect (ω^2^ = 0.034). Empathy and satisfaction with life had negligible effects (ω^2^ = 0.002).

Levene's test (Table 1) was conducted to confirm the assumption of homogeneity of variances across the levels of each independent variable. The results indicated *p-values* >0.05 for all variables, supporting the validity of the ANOVA analysis.

### 3.1 Group means and *post hoc* analysis of developmental assets

The means and standard deviations for each level of the independent variables are presented in Table 2. The charts displaying the results of the Games–Howell *post hoc* tests are included in the Supplementary material.

**Table 2:** Means, standard deviations, and standard errors for psychosocial adjustment across levels of developmental assets.

**Variable**	**Level**	**N**	**Mean**	**SD**	**SE**
Emotional repair	Low	43	32.19	9.49	1.45
	Medium	81	39.38	8.06	0.90
	High	45	45.40	7.85	1.17
Emotional clarity	Low	40	32.78	8.73	1.38
	Medium	85	38.65	8.10	0.88
	High	44	45.93	8.87	1.34
Attention to emotion	Low	40	31.25	9.58	1.51
	Medium	83	39.95	6.90	0.76
	High	46	44.59	9.60	1.42
Empathy	Low	51	37.96	9.09	1.27
	Medium	67	38.82	8.64	1.06
	High	51	40.78	11.20	1.57
Self-esteem	Low	30	35.57	10.99	2.01
	Medium	103	39.15	8.93	0.88
	High	36	42.17	9.54	1.59
Optimism	Low	48	34.23	10.10	1.46
	Medium	66	40.30	7.82	0.96
	High	55	42.07	9.67	1.30
Satisfaction with life	Low	43	38.51	10.18	1.55
	Medium	84	38.49	9.54	1.04
	High	42	41.14	9.12	1.41

For emotional repair, the mean scores for psychosocial adjustment were *M* = 32.19, *SD* = 9.49 for the low group, *M* = 39.38, *SD* = 8.06 for the medium group, and *M* = 45.40, *SD* =7.85 for the high group. *Post hoc* comparisons using Games–Howell tests revealed significant differences between the low and medium levels (*p* < 0.001), low and high levels (*p* < 0.001), and medium and high levels (*p* < 0. 001). Similarly, for emotional clarity, the low group obtained a mean score of *M* = 32.78, *SD* = 8.73, the medium group *M* = 38.65, *SD* = 8.10, and the high group *M* = 45.93, *SD* = 8.87. *Post hoc* tests indicated significant differences between the low and medium levels (*p* < 0.001), low and high levels (*p* < 0.001), and medium and high levels (*p* < 0.001). For attention to emotions, the mean scores were *M* = 31.25, *SD* = 9.58 for the low group, *M* = 39.95, *SD* = 6.90 for the medium group, and *M* = 44.59, *SD* = 9.60 for the high group. Significant differences were found between the low and medium levels (*p* < 0.001), low and high levels (*p* < 0.001), and medium and high levels (*p* < 0.05).

In the case of empathy, the low group had a mean score of *M* = 37.96, *SD* = 9.09, the medium group *M* = 38.82, *SD* = 8.64, and the high group *M* = 40.78, *SD* =11.20. *Post hoc* analyses showed no significant differences between the levels (*p* > 0.05). For self-esteem, the mean scores were *M* = 35.57, *SD* = 10.99 for the low group, *M* = 39.15, *SD* = 8.93 for the medium group, and *M* = 42.17, *SD* = 9.54 for the high group. Significant differences were observed only between the low and high levels (*p* < 0.05).

Regarding optimism, the mean scores were *M* = 34.23, *SD* = 10.10 for the low group, *M* = 40.30, *SD* = 7.82 for the medium group, and *M* = 42.07, *SD* = 9.67 for the high group. *Post hoc* tests revealed significant differences between the low and medium levels (*p* < 0.001) and the low and high levels (*p* < 0.001), but no significant differences between the medium and high levels (*p* > 0.05). Finally, for satisfaction with life, the low group reported a mean score of *M* = 38.51, *SD* = 10.18, the medium group *M* = 38.49, *SD* = 9.54, and the high group *M* = 41.14, *SD* = 9.12. *Post hoc* analyses did not reveal significant differences between the levels (*p* > 0.05).

The results indicate that higher levels of emotional repair, emotional clarity, attention to emotions, and optimism are strongly associated with improved psychosocial adaptation outcomes, with large to moderate effect sizes. The small effect size for self-esteem suggests a more limited but still significant contribution. The negligible effect sizes for empathy and satisfaction with life support their lack of significance in the ANOVA, reinforcing that these variables may have a limited role in explaining psychosocial adaptation in this population.

Although the one-factor ANOVA provided valuable insights into the effects of different levels of developmental resources on psychosocial adaptation, its scope is limited to testing mean differences in the dependent variable across predefined levels of the independent variables. To complement these findings, a MCA was performed. This approach allows for a more detailed exploration of the multivariate relationships among the categorical variables, enabling the identification of patterns and associations that go beyond the direct effects measured in the ANOVA.

### 3.2 Analysis MCA

The MCA was particularly useful in this study to visualize how different levels of developmental resources collectively interact and cluster in relation to Psychosocial Adaptation. This technique provides a descriptive framework to observe complex relationships and latent structures within the data, offering an additional perspective to understand the phenomenon holistically. By combining ANOVA and MCA, we were able to integrate inferential and exploratory analyses, thus ensuring a comprehensive understanding of how developmental resources contribute to psychosocial adaptation in socially vulnerable youth.

Therefore, for the execution of this analysis, Psychosocial Adaptation, as the dependent variable, was categorized into three levels: maladjustment, normal, and over-adaptation, based on the cut-off points specified in the Section 2.2. These levels were analyzed alongside the low, medium, and high levels of the developmental assets.

**Figure 1:**
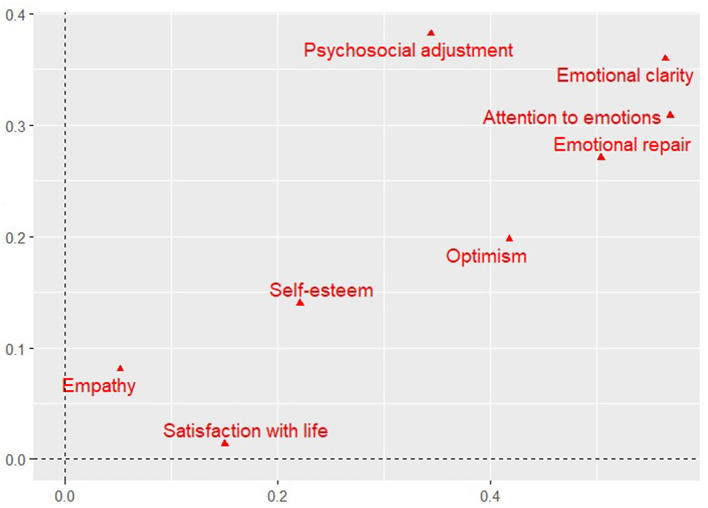
Model discriminant measures.

The computer algorithm needed 29 iterations to fit the model, in which two dimensions are proposed to explain 57.1% of the total variance, distributed as follows: dimension 1 (with a contribution of 35.2%) and dimension 2 (21.9%). Figure 1 shows the discrimination measures according to the variances obtained for each category (see Supplementary Table 2), indicating that variables with the greatest discriminative weight or that could better explain this model are those related to EI as assets of the socio-emotional development of adolescents, followed (with less discriminative value) by optimism and self-esteem as assets of personal development. Conversely, satisfaction with life and empathy did not show greater discriminative power in the model, suggesting that they cannot explain psychosocial adaptation in this population. According to the values of variances (see Supplementary Table 1) for each variable, factors associated with the assets of emotional and personal positive development fall into the first dimension, whereas psychosocial adaptation mainly lies in the second dimension. These MCA results seem to confirm the ANOVA results regarding the small effect that empathy and satisfaction with life seem to have on psychosocial adaptation.

**Figure 2:**
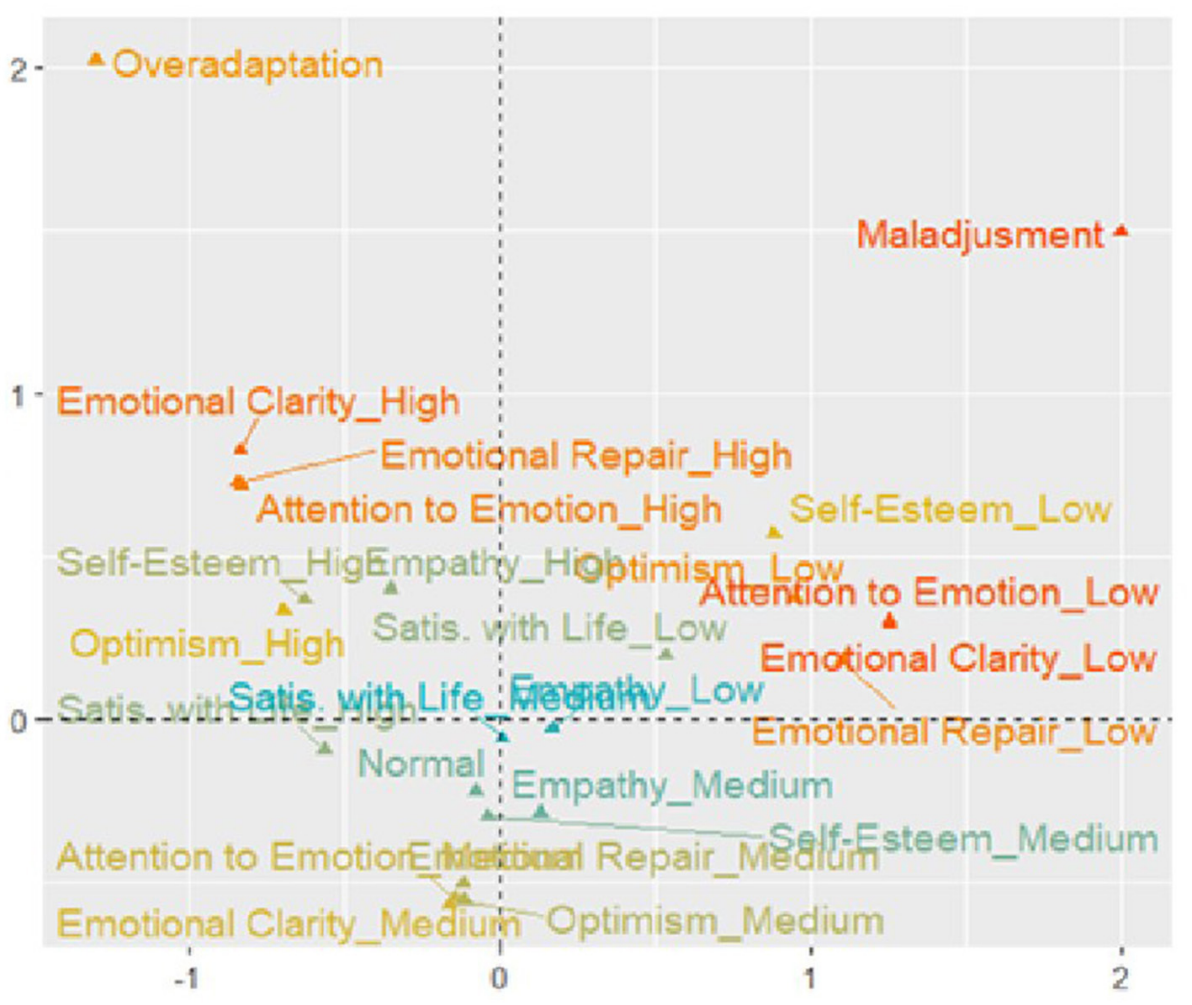
Point map and correspondence of variables and their categories.

Figure 2 presents the differences in the dispositions analyzed through the point map of the variable categories, showing that normal psychosocial adaptation is distributed in the lower quadrants with moderate or medium levels of emotional and personal developmental assets, whereas the pathological aspects of adaptation are in the upper quadrants. Therefore, maladjustment is perceived in the upper right quadrant, with low levels of self-esteem, optimism, satisfaction with life, and low levels of attention, emotional repair, and emotional clarity. Conversely, the upper left quadrant reveals an associational tendency between overadaptation and high levels of emotional and personal developmental assets.

## 4 Discussion

The present study aimed to empirically investigate the relationship between developmental assets and psychosocial adjustment in socially vulnerable youth, hypothesizing that personal and socio-emotional developmental asset would facilitate adaptive responses, and enhance psychosocial adjustment. Consistent with this hypothesis, the results of the one-factor ANOVA indicated significant effects of emotional repair, emotional clarity, and emotional attention on psychosocial adaptation, suggesting that higher levels of these socio-emotional assets are associated with better adaptation outcomes. This finding is consistent with the proposal that social-emotional resources play a critical role in fostering adaptive responses in adolescents (Prati and Pietrantoni, 2009) and further understands that the adaptive process requires not only connective skills, but also individual skills, as expressed by Palomera et al. (2012). A relevant finding, as previous literature has highlighted the need to expand studies that directly relate self-esteem to psychosocial adjustment and adaptation in the general population. Self-esteem has been shown to be associated with greater personal success and wellbeing, as well as health and occupational functioning in socially vulnerable and non-vulnerable contexts (Neely-Prado et al., 2019; Orth and Robins, 2014).

In contrast, satisfaction with life and empathy did not show significant effects on psychosocial adaptation, suggesting that these assets may have limited influence in the context of socially vulnerable youth. This is consistent with previous findings indicating that certain developmental resources may not uniformly contribute to adaptation across populations, and thus psychosocial adaptation may vary specifically in vulnerable contexts (Neely-Prado et al., 2021). It is therefore recommended that these assets be further studied in different contexts and populations, bearing in mind that empathy has been shown to be an emotional response that facilitates the understanding of social behavior (Mestre et al., 2004), and that life satisfaction depends on many factors that can affect the psychological wellbeing of young people, even in a vulnerable social context (Liberalesso, 2002).

The results of the MCA revealed that social-emotional assets, particularly emotional repair, clarity, and mindfulness, had the greatest discriminative power in explaining psychosocial adjustment. This supports the hypothesis that these factors are critical in shaping adaptive responses, i.e., the acquisition of essential competencies to identify and manage emotions, set and achieve positive goals, appreciate others' perspectives, maintain healthy relationships, make responsible decisions, and manage interpersonal situations constructively. They not only promote positive adjustment but also reduce risk factors in young people (Elias et al., 1997). Additionally, the observed clustering of maladjustment with low levels of developmental assets and over-adaptation with high levels reinforces the value of these resources in understanding adaptation patterns.

This study of adolescents' adaptation and psychosocial adjustment has allowed us to explore aspects related to the resources that should be recognized and promoted in educational and social contexts. In this sense, the meta-analysis conducted by Durlak et al. (2011) revealed that students in educational institutions in which social and emotional skills teaching programs were implemented showed a significant improvement in their socioemotional competencies, attitudes, behaviors and academic performance, a finding previously supported by the study of Snyder et al. (2009). Therefore, it would be pertinent to conduct a longitudinal intervention study to measure the effect of the implementation of an emotional literacy program integrated into the curriculum. In subsequent phases, this program could be expanded to involve both parents and teachers, promoting a comprehensive and sustainable approach to the development of socioemotional competencies in the socially vulnerable student population.

### 4.1 Limitations and future directions

The results of this study have important theoretical and practical implications in relation to individual and socioemotional assets in the psychosocial adjustment of adolescents in vulnerable social contexts. It has also clarified the mediating role of some emotional variables in psychological adjustment and personal wellbeing in contexts of social vulnerability and highlight the need, as pointed out by some authors, to optimize the socio-emotional development of adolescents through school, family and social contexts (Oliva et al., 2008), as well as the need for public policies that ensure investment in socio-educational programs for vulnerable children and adolescents in early stages of development, not only to promote the development of essential competencies for their positive development, but also to ensure their psychosocial adaptation and social and economic integration in adulthood (Heckman, 2008; Lin et al., 2024).

One of the limitations of the study is that the data collection was carried out in only one educational institution in the city of Cartagena—Colombia, however, in this institution the population of secondary school students was in conditions of social vulnerability due to factors of displacement by armed conflict and natural disaster. Future research should focus on replicating this study in vulnerable areas in other regions of Colombia to confirm the pattern of association suggested by MCA. Once this pattern has been identified and confirmed, it would be pertinent to develop a study that analyzes the mediating and moderating role of developmental assets and sociodemographic variables in the psychosocial adjustment of students. This approach would allow a deeper understanding of how these variables interact and contribute to the overall wellbeing of the student population in contexts of vulnerability. Likewise, it is relevant to design and carry out research aimed at evaluating the impact of extracurricular activities (artistic, musical, sports) as an educational strategy to promote the development of socio-emotional and personal competencies, as suggested by Portela-Pino et al. (2021). These activities could act as a key psychoeducational resource to strengthen positive development and adaptation in the school, family and social environment. Finally, possible lines of research are suggested to further investigate the levels of development of these resources. A moderate development of personal and socioemotional resources could lead to better adaptive responses and greater psychosocial adjustment in adolescents.

## 5 Conclusions

The present study aimed to empirically investigate the relationship between developmental assets and psychosocial adjustment in socially vulnerable youth. In this respect, the combined findings from ANOVA, *post hoc* testing, and MCA provided a comprehensive understanding of how developmental assets influence psychosocial adjustment. The results confirmed that higher levels of socio-emotional and personal developmental assets would be associated with better psychosocial adjustment. These findings add to research that has shown that adolescents who are more active show more positive and therefore healthy development (Benson et al., 2013). Conversely, the limited impact of empathy and life satisfaction suggested that these resources may not contribute significantly to adjustment in socially vulnerable adolescents.

## Data Availability

The raw data supporting the conclusions of this article will be made available by the authors, without undue reservation.
