# Supplementary material for: Socio-emotional and personal development competencies as assets facilitating psychosocial adaptation in socially vulnerable secondary school students
Source: Front Psychol. 2025 Apr 30;16:1462605. doi: 10.3389/fpsyg.2025.1462605 (PMC12077422; doi:10.3389/fpsyg.2025.1462605)
Supplement: Supplementary file 1 [file Data_Sheet_1.pdf]

Graphs with Games-Howell Analysis between levels of developmental assets and type of social adaptation

Socio-emotional development

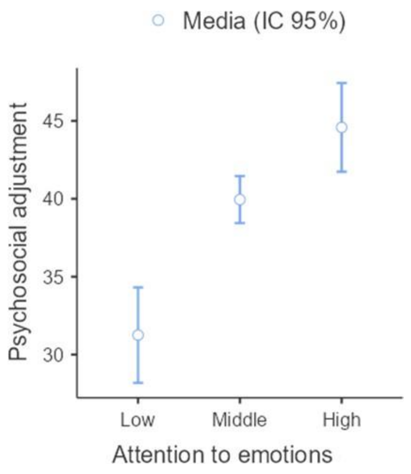

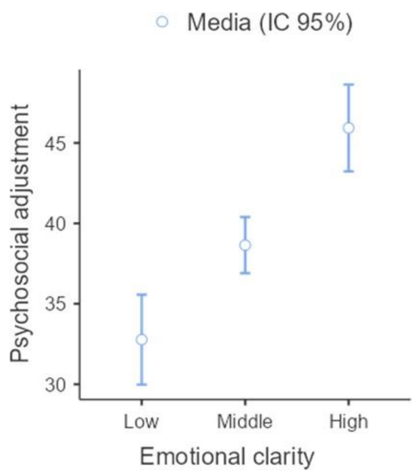

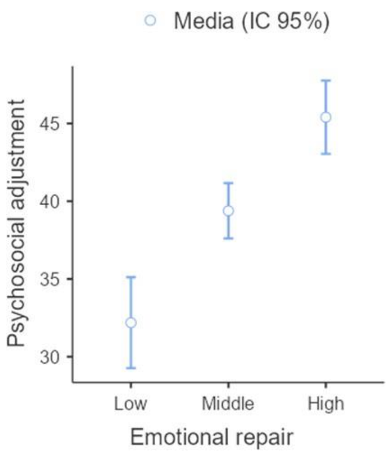

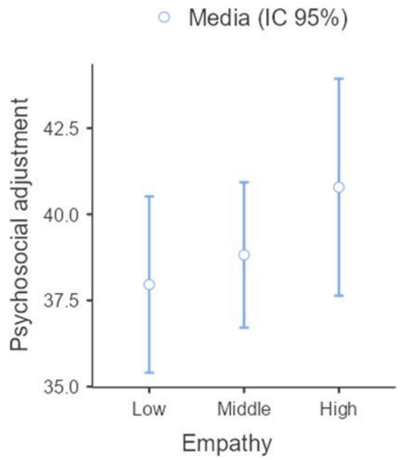

Personal development

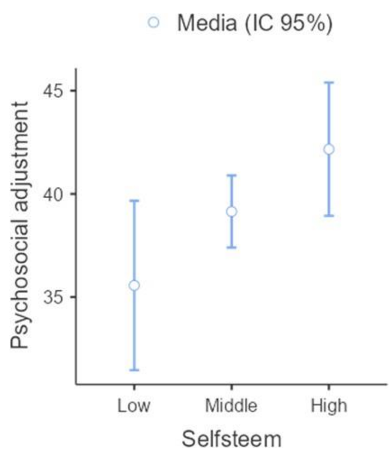

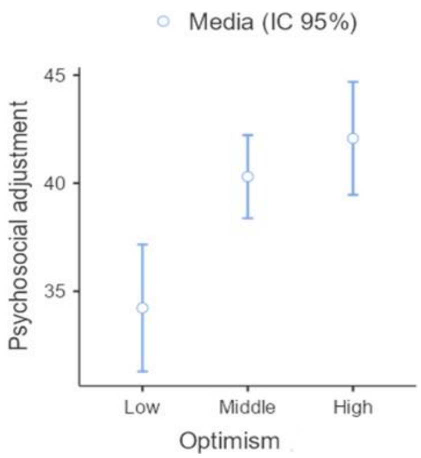

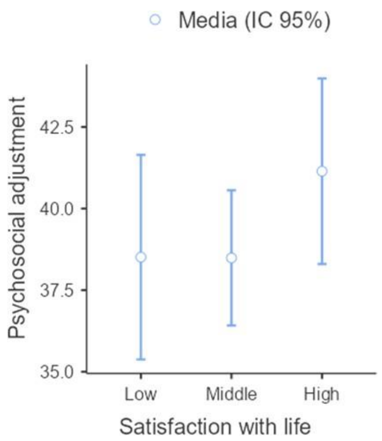

Supplementary table 1. Scores Based on the Cut-Off Point for Each Scale of Developmental Assets

TMMS-24 scores based on cut-off points derived from the scale's percentiles (Oliva et al., 2011).

Dimension	Gender	Description	Score Range
**Attention to emotions**	Men	Low: Needs to improve attention: pays little attention	< 21
**Attention to emotions**	Men	Medium: Adequate attention	22 to 32
**Attention to emotions**	Men	High: Needs to improve attention: pays too much attention	> 33
**Emotional clarity**	Men	Low: Needs to improve emotional clarity	< 25
**Emotional clarity**	Men	Medium: Adequate emotional clarity	26 to 35
**Emotional clarity**	Men	High: Excellent emotional clarity	> 36
**Emotion repair**	Men	Low: Needs to improve emotion repair	< 23
**Emotion repair**	Men	Medium: Adequate emotion repair	24 to 35
**Emotion repair**	Men	High: Excellent emotion repair	> 36
**Attention to emotions**	Women	Low: Needs to improve attention: pays little attention	< 24
**Attention to emotions**	Women	Medium: Adequate perception	25 to 35
**Attention to emotions**	Women	High: Needs to improve attention: pays too much attention	> 36
**Emotional clarity**	Women	Low: Needs to improve emotional clarity	< 23
**Emotional clarity**	Women	Medium: Adequate emotional clarity	24 to 34
**Emotional clarity**	Women	High: Excellent emotional clarity	> 35
**Emotion repair**	Women	Low: Needs to improve emotion repair	< 23
**Emotion repair**	Women	Medium: Adequate emotion repair	24 to 34
**Emotion repair**	Women	High: Excellent emotion repair	> 35

Rosenberg Self-Esteem Scale. Scores based on cut-off points derived from the scale's percentiles (Gomez-Lugo et al., 2016)

Gender	Low (< 25th Percentile)	Medium (25th to 75th)	High (> 75th Percentile)
Women	30	31-36	37
Men	31	32-36	37

Optimism (General Mood subscale). Scores based on cut-off points derived from the scale's percentiles (Oliva et al., 2011).

Age/Gender group	Low (< 25th Percentile)	Medium (25th to 75th)	High (> 75th Percentile)
14-15 years (Female)	28	29-36	37
14-15 years (Males)	30	31-38	39
16 + years (Female)	27	28-34	35
16 + years (Males)	29	30-37	38

Student's Life Satisfaction Scale. Scores based on cut-off points derived from the scale's percentiles (Oliva et al., 2011).

Age/Gender group	Low (< 25th Percentile)	Medium (25th to 75th)	High (> 75th Percentile)
14-16 years (Both genders)	32	33-41	42
16 + years (Females)	29	30-40	41
16 + years (Males)	32	33-41	42

The Basic Empathy Scale. Scores based on cut-off points derived from the scale's percentiles (Oliva et al., 2011).

Age/Gender group	Low (< 25th Percentile)	Medium (25th to 75th)	High (> 75th Percentile)
14-15 years (Females)	33	34-38	39
14-15 years (Males)	28	29-34	35
16 + years (Females)	33	34-37	38
16 + years (Males)	29	30-34	35

Supplementary table 2. Discriminant measures MCA

	Variable weighting	Dimension 1	Dimension 2	Mean
Optimism	3	,418	,199	,308
Empathy	3	,052	,082	,067
Satisfaction with life	3	,151	,013	,082
Self-esteem	3	,221	,140	,181
Emotional repair	3	,504	,271	,387
Emotional clarity	3	,564	,358	,461
Attention to emotions	3	,569	,307	,438
Psychosocial adjustment	3	,344	,382	,363
Total assets [a]	24	8,466	5,257	6,862
% variance		35,277	21,905	28,591

a. The variable weights have been incorporated in the statistics of total assets
